# Coal-gangue recognition via multi-branch convolutional neural network based on MFCC in noisy environment

**DOI:** 10.1038/s41598-023-33351-4

**Published:** 2023-04-21

**Authors:** HaiYan Jiang, DaShuai Zong, QingJun Song, KuiDong Gao, HuiZhi Shao, ZhiJiang Liu, Jing Tian

**Affiliations:** 1 Department of Intelligent Equipment, Shandong University of Science & Technology, Taian, 271000 China; 2 Shandong Province Key Laboratory of Mine Mechanical Engineering, Shandong University of Science & Technology, Qingdao, 266590 China; 3 Hong Kong Baptist University, Hong Kong, China; 4 Taihe Electric Power Co. Ltd, Taian, 271000 Shandong China

**Keywords:** Engineering, Mathematics and computing

## Abstract

Traditional coal-gangue recognition methods usually do not consider the impact of equipment noise, which severely limits its adaptability and recognition accuracy. This paper mainly studies the more accurate recognition of coal-gangue in the noise site environment with the operation of shearer, conveyor, transfer machine and other device in the process of top coal caving. Mel Frequency Cepstrum Coefficients (MFCC) smoothing method was introduced to express the intrinsic feature of sound pressure more clearly in the coal-gangue recognition site. Then, a multi-branch convolution neural network (MBCNN) model with three branches was developed, and the smoothed MFCC feature was incorporated into this model to realize the recognition of falling coal and gangue in noisy environment. The sound pressure signal datasets under the operation of different device were constructed through a great deal of laboratory and site data acquisition. Comparative experiments were carried out on noiseless dataset, single noise dataset and simulated site dataset, and the results show that our method can provide higher correct recognition accuracy and better robustness. The proposed coal-gangue recognition approach based on MBCNN and MFCC smoothing can not only recognize the state of falling coal or gangue, but also recognize the operational state of site device.

## Introduction

In China, fully mechanized top coal caving technology is one of the important mining technologies in coal mine production, and coal-gangue recognition is the key to realize the intellectualization of fully mechanized top coal caving. The realization of intelligent coal caving process can not only solve the over-caving and less-caving problem, improve the quality and efficiency of mining, but also reduce the impact of the adverse environment on the health of workers. State recognition system is defined from the perspective of pattern recognition, usually including three steps: data acquisition (DAQ), feature extraction and pattern recognition, among which, the latter two are of great significance and have a great impact on the accuracy of the final recognition. The research on DAQ of coal-gangue recognition can be traced back to the 1960s, mainly including natural gamma ray^1,2^, image recognition^3,4^, sound signal analysis^5^, vibration signal analysis^6–8^, sound and vibration signal analysis^9–11^. Natural gamma ray method requires that there must be a lot of radioactive elements in coal and gangue, and the cost of sensor is high; Image recognition is greatly affected by on–site dust, so it is difficult to obtain an ideal image of coal-gangue in caving; Sound and vibration signal acquisition is simple, which is a common method at present. However, sound signals are mixed with noise from different field equipment operations, which makes accurate identification of sound signals more difficult than vibration signals. Therefore, how to accurately achieve automatic target recognition of sound provides important technical support for the realization of intelligent fully mechanized top coal caving.

At present, the methods of feature extraction mainly include Hilbert–Huang Transform (HHT)^6,7,10^, wavelet packet transform^11^, Mel Frequency Cepstrum Coefficients (MFCC)^5,9^, time domain feature extraction8 and other variants. By using Mel scale based on the sensitivity of the human ear, MFCC can provide better results than the operations in time domain^12^, which is commonly used for feature extraction in the frequency domain for different sound-based applications^13^. Su et al. combined log-Mel spectrogram and MFCC to complete the feature extraction of urban noise in Urbansound8K dataset, and designed a four-layer convolution neural network (CNN) based on DS theory, and indicated that the proposed method is suitable for environmental sound classification^14^. Rujoie et al. used MFCC and wavelet transform (WT) to obtain the better features of sound signal generated by the heart, and gave the diagnosis and determination of the severity of tricuspid regurgitation with K-nearest neighbors (KNN) classifier, which provided a basis for the early diagnosis of cardiovascular diseases^15^. Bharath et al. proposed a speaker recognition method in noisy environment based on MFCC and power normalized cepstrum coefficient (PNCC) technology with fusion strategy^16^. Jung et al. used short-time Fourier transform and MFCC to extract the features of lung sounds, revealed the relationship between lung sounds and pulmonary mechanism, and employed the depth separable CNN to effectively classify four types of lung sounds^17^. Nasef et al. reported a recognition technique to distinguish gender using MFCC features and Logistic Regression (LG) classifier, which can be carried out in the presence of background noise and different language, accent, age and emotional states^18^. It can be seen that MFCC is a powerful method to represent intrinsic characteristics of the sound signals. Therefore, we also use MFCC to distinguish the sound induced by the impact of top coal and gangue on the hydraulic support.

The above sound recognition method directly processed the features after noise reduction, but did not make full use of the complementary information between the original features and the denoised features. In order to improve the accuracy of target recognition, scholars have proposed a multi branch convolutional neural network. For the sake of recognition accuracy improvement of health conditions of wind turbine gearbox, Jiang et al*.* established a multi-scale CNN model with three branches^19^, which can simultaneously extract and classify multi-scale features, obtain complementary rich features, effectively suppress noise components, and achieve higher diagnostic result. Considering the features of license plate’s small size and vehicle’s large as well as various size, Chen et al*.* proposed an end-to-end two multi-branch network with different convolutional layers^20^, which can effectively solve the problem of simultaneous detection of vehicle and license plate. A MBCNN model based on synchro-squeezed wavelet transform was reported^21^, which had rich transient stress wave properties for dynamic rail cracks. It can effectively solve the rail crack monitoring of complex crack conditions and high operational noise in the field, and get higher accuracy than the traditional CNN model. In order to solve the feature deficiency of a single bearing fault signal, Wang et al*.* proposed to bring multi-scale average processing into MBCNN^22^, which can obtain complementary feature information from multi-scale reconstructed signals and realize bearing fault diagnosis under different working conditions. In addition, Zuo et al. proposed a multi-branch 3D CNN model to achieve the detection of different shapes and sizes of nodules and non-nodules in lung^23^. Each branch of the model processed feature mapping from different depth layers, which can effectively reduce false positives in the detection of lung nodules. In short, the MBCNN can solve the feature deficiency of single signal, provide complementary and rich features, effectively suppress noise components, and improve recognition accuracy.

In the process of top-coal caving, the collected sound signal includes not only the sound of coal or gangue to be recognized, but also the noise induced by the operation of conveyor, shearer and other multiple device. MFCC features have strong robustness and are less affected by background noise, and multi-branch CNN has the ability to process different features independently. Inspired by these ideas, this paper proposes a new multi-branch CNN architecture with MFCC smoothing to recognize the state of top coal running under noise environment. The remainder of this paper is organized as follows. “Proposed models” section briefly reviews MFCC feature extraction and the basic process of CNN for target recognition. “Proposed MBCNN for coal-gangue recognition based on MFCC” section describes in detail the proposed MBCNN framework for coal-gangue recognition based on MFCC. “Experimental setup and performance evaluation index” section sets up an experimental platform to simulate the top coal caving on site and gives the performance evaluation index. “Experimental results and analysis” section evaluates the performance of the proposed method on various experimental datasets. Finally, a brief conclusion is provided in “Conclusions” section.

## Proposed models

The sound recognition method for controlling top coal caving was proposed in this study based on MFCC and MBCNN. It mainly consists of two stages. The first stage is the preprocessing stage, which uses MFCC to obtain more accurate feature representation of sound wave induced by top coal hitting different device. The second stage is the recognition stage of coal-gangue, which uses MBCNN to achieve higher recognition accuracy under various working environments.

### Preprocessing stage

**Figure 1:**
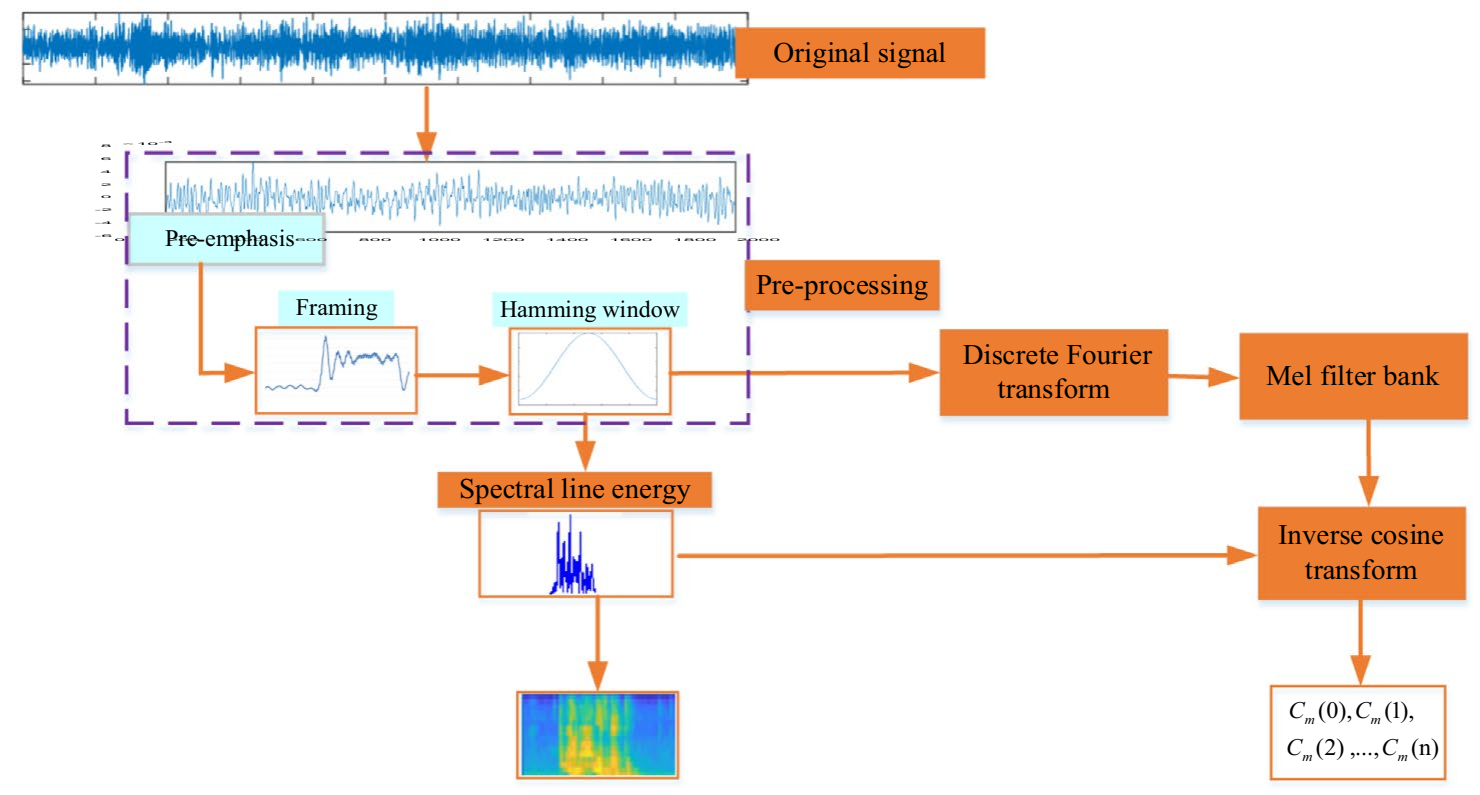
Schematic of MFCC feature extraction.

MFCC is the coefficient of the short-time windowed signal obtained by fast Fourier transformation (FFT), which has better results than the time domain operation. MFCC feature extraction mainly includes six steps^18^: pre-weighting, framing, windowing, FFT, Meyer filter bank and discrete cosine transform (DCT), as shown in Fig. 1. In this work, each sound file took 4 s at a sampling rate of 22 kHz, the length of each frame is 2048, and the frame shift is 512.

**Figure 2:**
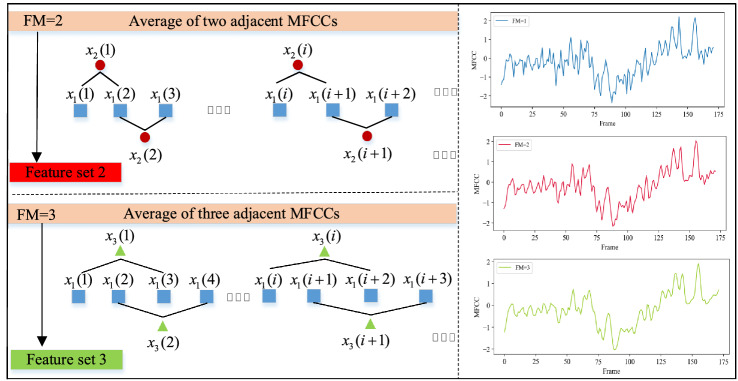
Description of MFCC feature smoothing processing method (FM = 2, 3).

In order to obtain more abundant features that can represent different sound classes, feature processing technique for sample expansion has been implemented. Perform MFCC feature extraction of sound signal using Fig. 1 to form the original feature set, called Feature Set 1 (FM = 1). In addition, the two adjacent frames in Feature Set 1 are averaged to form a new Feature Set 2 (FM = 2), and the three adjacent frames in Feature Set 1 are averaged to form a new Feature Set 3 (FM = 3). Taking the first order of the MFCC feature matrix of a coal signal as an example to illustrate the feature smoothing idea, it is assumed that the feature sequence of the first order is $x_{1} (k)$,* k* is the frame sequence number,* k* = 0,1,2,… 188. Figure 2 describes the processing process and results of Feature Sets 2 and 3.

### Multi-branch convolutional neural network

In this work, the core of coal-gangue recognition is represented by analyzing the difference of sound generated by the top coal caving impacting different device. For their implementation, we design a multi-branch CNN structure for coal-gangue recognition system.

### MBCNN architecture

**Figure 3:**
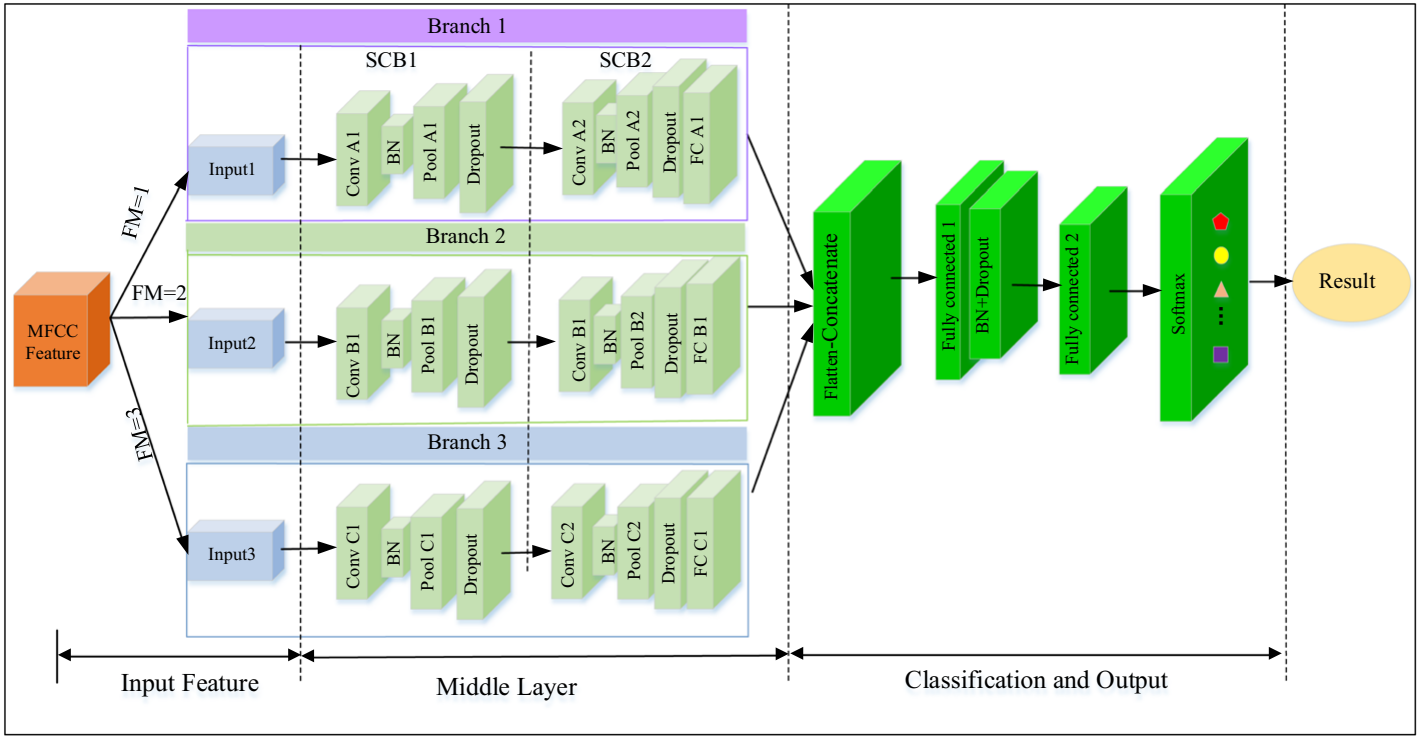
Proposed MBCNN framework for coal-gangue recognition using MFCC feature.

This paper focuses on the state recognition of falling coal or gangue in the process of top coal caving. In such case, the sound signals measured in different states are greatly affected by noise, which further challenges the separability of simply extracting state classification features using traditional CNN network structure. In order to make full use of the MFCC distribution characteristic of coal-gangue sound signal, a MBCNN model with three branches was proposed, as shown in Fig. 3.

The model mainly includes input feature, middle layer and classification. The features of the first part are derived from the three feature sets (Feature set *i*, *i* = 1,2,3) in Fig. 1, which provide complementary features for the recognition of coal-gangue and are respectively input into corresponding CNN branches. The second part is the middle layer, which consists of three branches, each of which has a different convolution kernel size to obtain the feature information under different receptive fields. A branch is composed of two standard convolution blocks (SCBs), and connected to the backbone layer respectively. Each branch separately processes different MFCC features, and then performs feature fusion through the full connectivity layer to obtain different coal-gangue features. Finally, a softmax classifier is used to complete state recognition.

### Training algorithm of CNN model

In the process of forward propagation, a convolutional neural network is mainly composed of five components: input layer, convolution layer, pooling layer, full connection layer and output layer.

CNN model is a supervised learning model, which needs to be learned under the supervision of sample tags. Therefore, the input data consists of sample *X* and sample label* Y*. For a *C* classification problem, the input of the model is represented by $\left\{ {X,Y} \right\} = \left\{ {x_{i} ,y_{i} } \right\}^{N}$, where *N* represents the number of samples of the input model, *x*_*i*_ represents the *i*-th sample, and *y*_*i*_ represents the class label corresponding to the *i*-th sample.

The convolution layer is the core component of CNN model, which realizes the idea of local connection and weight sharing through the convolution kernels (also named learnable filters). The convolution operation extracts features from the input feature map according to the convolution kernel size and the moving step size. The feature extraction process of convolution kernel is defined by

1 $${\mathbf{x}}_{j}^{L} = f(\sum\limits_{{i \in M_{j} }} {{\mathbf{w}}_{i,j}^{L} * {\mathbf{x}}_{i}^{L - 1} + {\mathbf{b}}_{j}^{L} } )$$

where $w_{i,j}^{L}$ and $b_{j}^{L}$ represent the weight matrix and bias matrix of the *j*-th convolution kernel in the *L*-th convolution layer, respectively. ${\mathbf{x}}_{i}^{L - 1}$ represents the *i*-th feature map output by the *L*-1 convolution layer and ${\mathbf{x}}_{j}^{L}$ represents the* j*-th feature map in the L convolution layer.$M_{j}$ is the number of convolution kernels. $f( \cdot )$ represents the nonlinear activation function. Here, the rectified linear units (ReLU)^24^ is used as the nonlinear activation function, as it is an unsaturated nonlinear function, ensuring that all outputs are nonnegative.

The full-connected layer connects each neuron in the previous layer and the next layer to flatten the feature maps into one vector. In order to improve the performance of CNN model, here the activation function of neurons in the full-connected layer adopts ReLU function, so the output of each neuron is expressed as:

2 $$z_{j}^{L} = {\text{ReLU}} (\sum\limits_{i = 1}^{M} {{\mathbf{w}}_{i,j}^{L} * {\mathbf{x}}_{i}^{L - 1} + {\mathbf{b}}_{j}^{L} } ),\;\;j = 1,2,...,N$$

where *M* and* N* represent the number of neurons in layer* L*-1 and layer *L*, respectively.

### Optimization method of CNN model

BN is an optimization method proposed by Sergey Ioffe^25^, a researcher at Google. By calculating the mean and variance estimates in the mini-batch of training sets, it adjusts the scale of input features, speeds up the training process of the model and enhances the generalization ability of the network. The BN layer is usually added after the convolution layer or full connection layer and before the activation unit. The BN layer output is computed with the input moment ${\hat{\mathbf{x}}}_{j}$ normalized by the second variable $\sigma^{L}$^25,26^:

3 $${\mathbf{y}}_{j}^{L} = c{\hat{\mathbf{x}}}_{j}^{L - 1} + d$$

4 $${\hat{\mathbf{x}}}_{j}^{L - 1} { = }\frac{{x_{j}^{L} - \overline{x}^{L} }}{{\sqrt {(\sigma^{L} )^{2} + \varepsilon } }}$$

where *c* and *d* are the scale factor and displacement factor respectively, which can be learned and adjusted during the training process.${\mathbf{x}}_{j}^{{L{ - }1}}$ is the* j*-th input in the *L*-1 layer. $\overline{x}^{L}$ and $\sigma^{L}$ are the mean and variance of the mini-bath in the *L*-1 layer respectively. $\varepsilon$ is a constant for numerical stability, and $\varepsilon \le 1$.

## Proposed MBCNN for coal-gangue recognition based on MFCC

**Figure 4:**
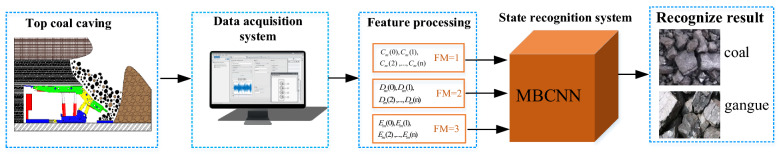
Technical route for identifying coal gangue under top coal.

In this section, we apply the proposed pattern recognition method based on MFCC and MBCNN to different fully mechanized mining faces to achieve intelligent recognition of coal-gangue. The technical route of this method is shown in Fig. 4, and the working procedures are briefly described as follows:

1. During the process of top coal caving, the data acquisition system independently developed by LabVIEW software is used to collect the sound pressure signals of coal and gangue in different fully mechanized mining faces, as well as on-site equipment such as shearer, conveyor and conveyor.
2. MFCC features are acquired by discrete cosine transform of log-Mel spectral energy, and the feature average processing is performed for the purpose of obtaining local information of coal gangue itself.
3. The constructed MBCNN state recognition system is used to learn useful features from original features and multiple reconstructed features, and the recognition results of different states under top coal are provided.

## Experimental setup and performance evaluation index

In this section, the construction of the experimental platform is described, and the performance evaluation criteria for the coal-gangue recognition system are given.

### Experimental setup

**Figure 5:**
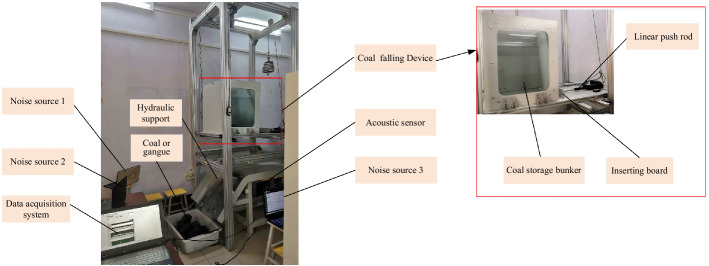
Schematic diagrams of the scaled physical model of top coal caving.

The experimental platform of top coal caving is mainly composed of coal falling device, hydraulic support, acoustic sensor, data acquisition system, and noise source (such as: shearer, conveyor, transfer machine), as shown in Fig. 5. Above the hydraulic support is the falling coal device, which is composed of coal storage bunker, inserting board and linear push rod. The inserting board can achieve the opening and closing of the coal caving window under the action of the linear push rod, and the working process of top coal caving is simulated. Sound signal is generated when coal or gangue impacts the tail beam of hydraulic support, and its frequency is 500–4000 Hz. The sound data is collected by HSL-10 sound sensor, whose working bandwidth is relatively stable in the range of 200–10,000 Hz, which can provide accurate data for coal–gangue recognition later.

### Evaluation criteria

Our task is to recognize the sound generated by top coal caving and reduce the impact of noise. Therefore, the confusion matrix method is used to evaluate the model. Confusion matrix is an effective tool for multi target recognition system^27^, which shows the comparison between output class and target class. The model evaluation based on the confusion matrix is given in Table 1. The output class is the recognition result of the model, and the target class is the real class.

**Table 1:** Model evaluation based on binary confusion matrix.

Confusion matrix		Actual value	
		Positive	Negative
Recognition value	Positive	True positive (TP)	False positive (FP)
	Negative	False negative (FN)	True negative (TN)

In order to evaluate the performance of the proposed method, the accuracy, precision and F1-score are used in^28^:

5 $$\text{Accuracy} = \frac{\text{TP} + \text{TN}}{{\text{TP} + \text{FP} + \text{FN} + \text{TN}}}$$

6 $$\text{Precision} = \frac{\text{TP}}{{\text{TP} + \text{FP}}}$$

7 $$\text{F1-score} = \frac{2\text{TP}}{{2\text{TP} + \text{FP} + \text{FN}}}$$

## Experimental results and analysis

### Experimental data and model description

Extensive experiments conducted on the coal-gangue recognition in top coal caving prove the superior performance of MBCNN. There are seven different sound classes in the coal-gangue dataset, including the noise induced by the device operation of the front conveyor, the rear conveyor, the right cutting of the shearer, the left cutting of the shearer and the transfer machine, as well as the state sounds of falling coal and gangue. Table 2 gives a detailed description of state labels for noiseless dataset.

**Table 2:** Description of noiseless dataset for coal-gangue recognition.

States label	Audio event class	States label	Audio event class
0	Falling coal	4	Transfer machine
1	Falling gangue	5	Right cutting of shearer
2	Rear conveyor	6	Left cutting of shearer
3	Front conveyor		

In this study, feature smoothing method of MFCC is adopted in MBCNN model. In order to illustrate the impact of this method on coal-gangue recognition performance, single-branch and double-branch CNN models are introduced, and their main structural parameters are described as follows:

- Model 1: Single-branch CNN model is used with Conv = 256, Kernel size = 6, Dense (10) + BN.
- Model 2: There is a double-branch CNN model with Conv = 256, Kernel sizes = 5 + 6, and Dense (10) + BN.
- Model 3: There are three branches of CNN proposed in this paper, Conv = 256, Kernel sizes = 4 + 5 + 6, and Dense (64) + Dense (32) + BN.

For Model 1, the Feature Set 1 is input into the convolutional neural network; For Model 2, the Feature Set 1 is input into the branch where Kernel size = 6, and the smoothed Feature Set 2 is input into the branch where kernel size = 5; For Model 3, the first two branches are consistent with Model 2, and the smoothed Feature Set 3 is input on the kernel size = 4 branch.

The performance of coal-gangue recognition of the above three models in the process of top coal caving under different conditions will be illustrated in the following section. In addition, the initial learning rate of Adam optimizer was 0.0001, the number of epochs was 200, and the batch size was 64.

### Experiment with noiseless dataset

**Figure 6:**
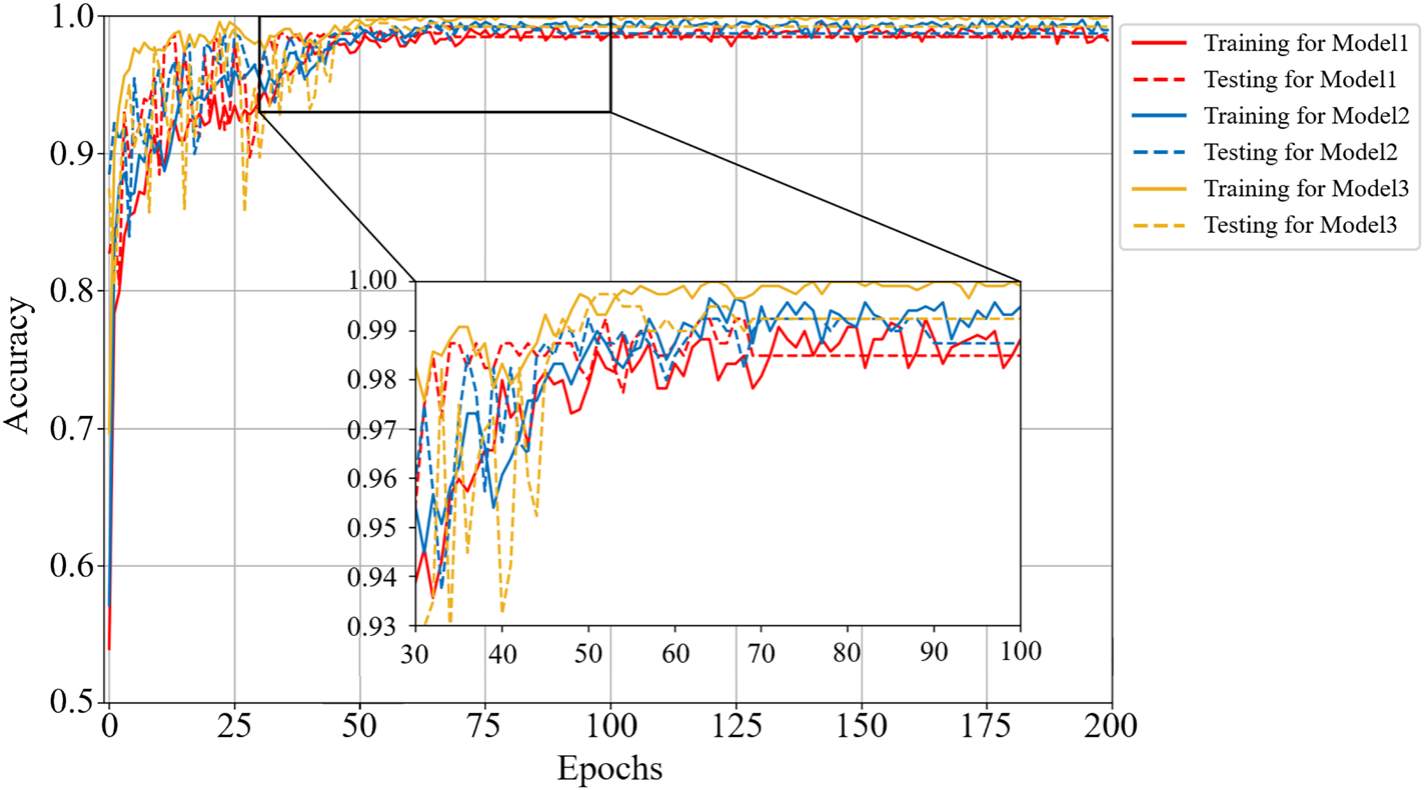
Comparison curves of accuracy performance of different models on noiseless dataset.

**Figure 7:**
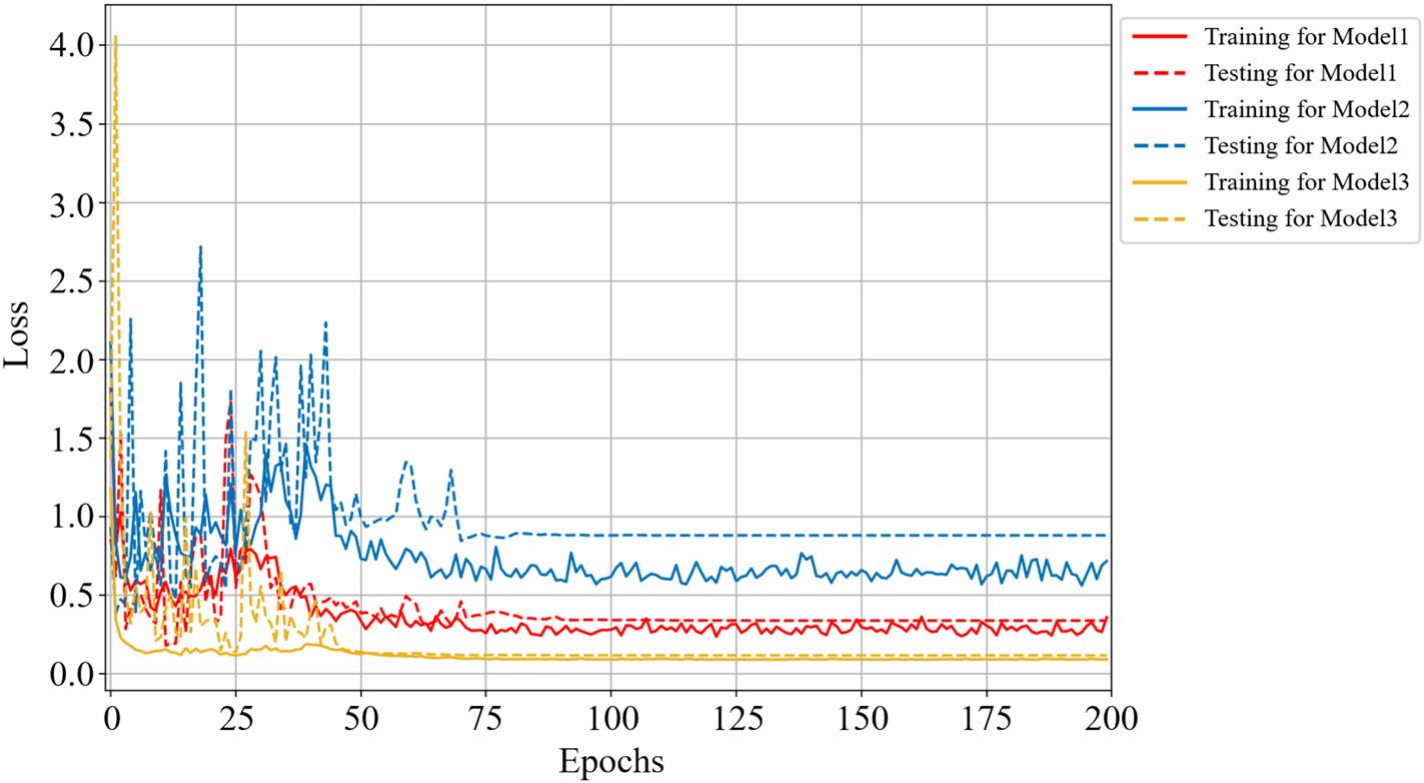
Comparison curves of loss performance of different models on noiseless dataset.

In this section, we discuss the effectiveness of the MBCNN model for noiseless dataset on the coal-gangue recognition. Firstly, Figs. 6 and 7 respectively show the accuracy and loss curves of the training set and the testing set for different CNN models.

For Model 1, the training accuracy and the testing accuracy reach the stable values after 69 epochs, and the testing accuracy is 98.50%. For Model 2, the training accuracy and the testing accuracy reach the stable values after 90 epochs, and the testing accuracy is 98.74%. For Model 3, the training accuracy and the testing accuracy reach the stable values after 68 epochs, and the testing accuracy is 99.28%. So, Model 3 converges to the stable value faster than the other two models, which means that training the Model 3 requires less time in practice. It is obvious from Fig. 7 that the loss function of Model 3 is smaller than that of the other two models, and the curve oscillation is the smallest before convergence, indicating that the robustness of Model 3 is the best on the noiseless dataset.

**Figure 8:**
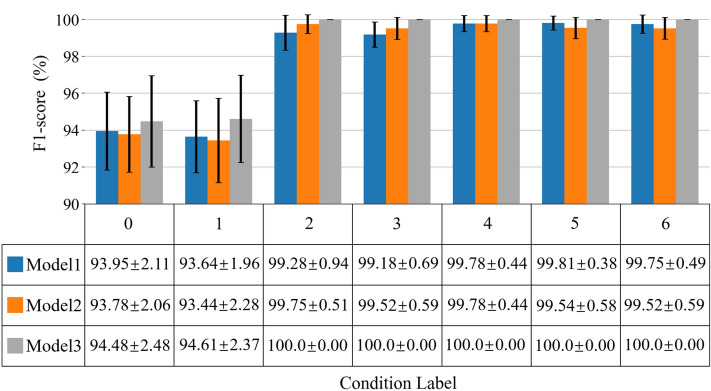
F1-score performance index of different models on noiseless dataset.

Then, the mean of F1-score for each class obtained through fivefold cross validation is shown in Fig. 8, where the error bar represents the standard deviation of the stability of recognition performance. By comparing the recognition effects of the three models on 7 kinds of sound signals, it is found that Model 3 can fully recognize the labels 2–6, while the other two models cannot completely identify a certain class state, and the recognition result of Model 3 is superior to the other two models in the recognition state of labels 0–1. In addition, in the case of labels 2–6, the standard deviations of Model 3 are all zero, indicating that it has a more reliable and stable performance. The results show that the proposed approach can learn the linkage features from different branches, so as to obtain higher quality recognition performance.

In a word, the proposed MBCNN model of three branches makes full use of the MFCC feature distribution of falling coal or gangue on different time–frequency scales, and has higher sound recognition accuracy than the traditional single branch CNN and double-branch CNN on most class labels.

### Site simulation experiment

**Figure 9:**
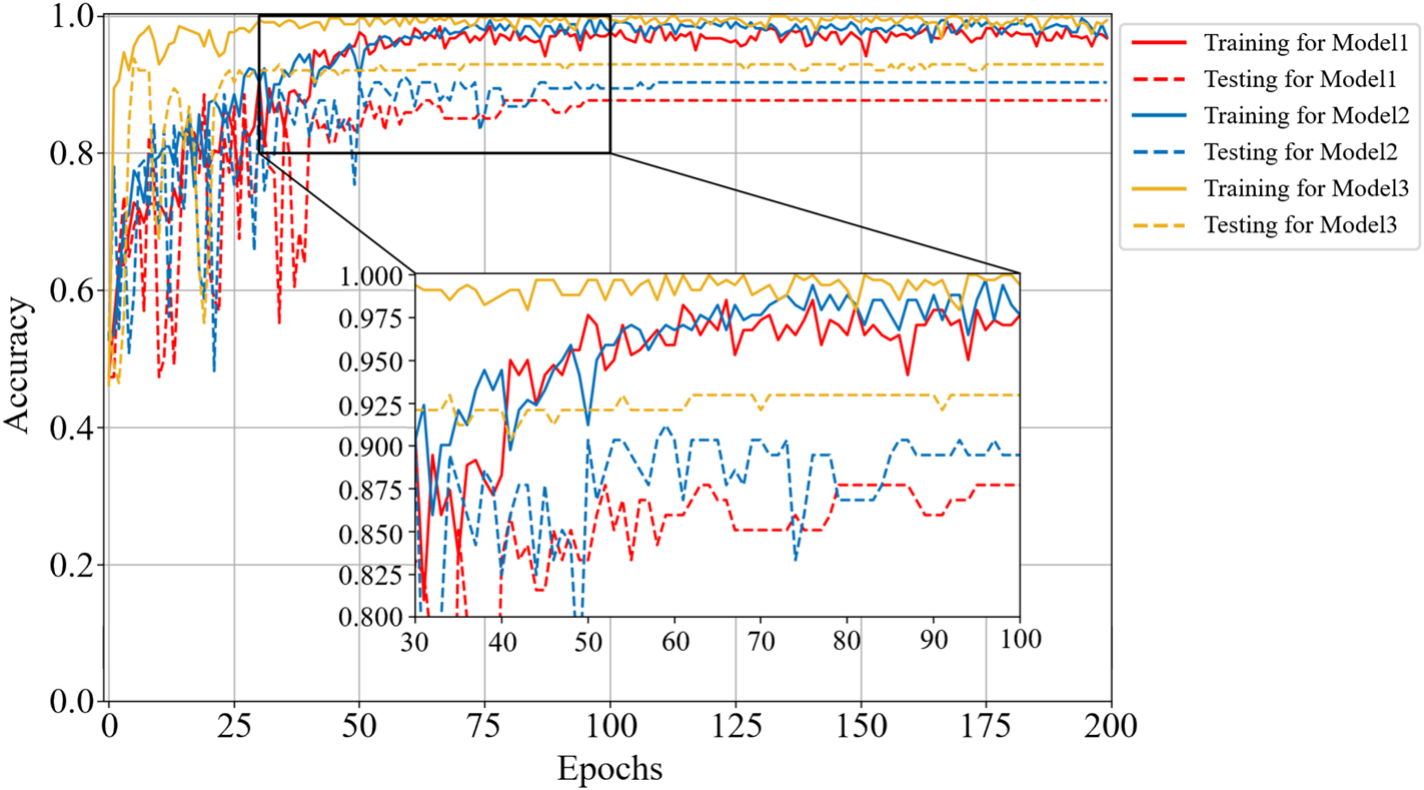
Comparison curves of accuracy performance of different models on simulated site dataset.

**Figure 10:**
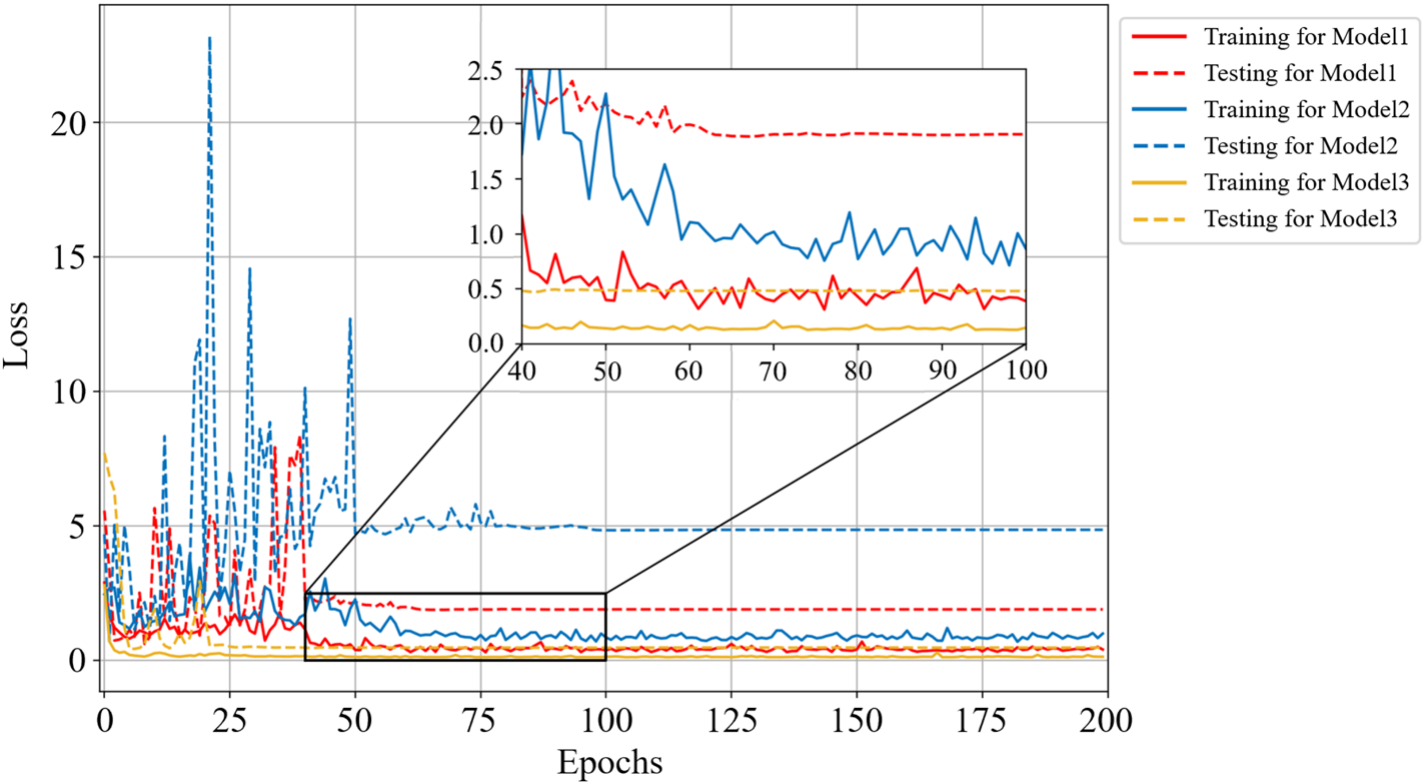
Comparison curves of loss performance of different models on simulated site dataset.

**Figure 11:**
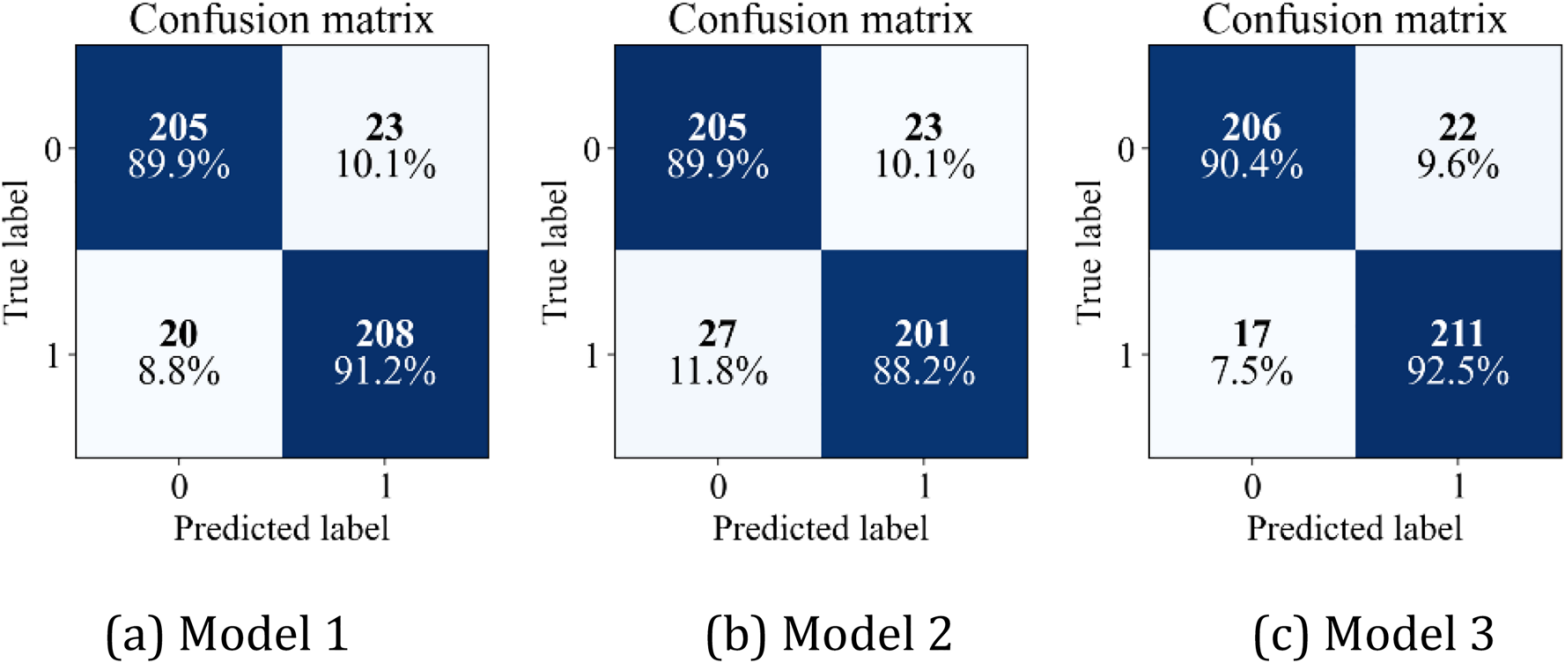
Confusion matrixes depicting the precision index on simulated site dataset.

In order to further verify the generalization ability of MBCNN in coal-gangue recognition during the process of top coal caving, according to Fig. 5, the coal and gangue caving as well as various noises were simultaneously mixed to simulate the site process of top coal caving to create a simulated site dataset. The simulated site dataset has two classes of labels, label 0 represents the sound of falling gangue, and label 1 represents the sound of falling coal. The difference from the previous research^5,9–11^ was that these two classes signals were collected when the rear conveyor, the front conveyor, the transfer machine, the shearer right cutting and the shearer left cutting were running at the same time, that is, noise induced by the operation of site device was taken into account. The curves of accuracy and loss function are shown in Figs. 9, 10, and the corresponding confusion matrixes are shown in Fig. 11.

As can be seen from Fig. 9, the testing accuracy of Model 1 reaches stable convergence after 96 epochs, and its convergence accuracy is 87.72%; the testing accuracy of Model 2 reaches stable convergence after 110 epochs, and its convergence accuracy is 90.35%; however, the testing accuracy of Model 3 reaches stable convergence only after 63 epochs, and its convergence accuracy is 92.98%. The loss function in Fig. 10 shows that Model 3 has the fastest convergence speed and the least oscillation amplitude change. Figure 11 depicts that the correct recognition rate of Model 1 and Model 2 in recognizing the falling coal is 89.9%, while the correct recognition rate of Model 3 in this class is 90.4%, and the correct recognition rate of Model 3 is the highest in the falling gangue.

On the simulation site dataset, the convergence accuracy of Model 3 is improved by 6.0% compared with Model 1, and 2.9% compared with Model 2, and Model 3 converges to the stable value faster than Model 1 and Model 2. At the same time, under the state of gangue caving, the recognition accuracy rate of Model 3 is 1.4% higher than that of Model 1, and 5.1% higher than that of Model 2. The results show that Model 3 has a better recognition accuracy ratio and requires less time in the practice of coal-gangue recognition. This is mainly because the feature smoothing processing method of three branches can effectively capture useful state feature information, so that Model 3 has better recognition performance in noisy environment.

### Comparison with traditional classification algorithm

In order to prove the advantages of the proposed MFCC-MBCNN, we compared several traditional feature extraction and classification algorithms, including Hilbert–Huang Transform (HHT) combined with bimodal deep neural networks (DNN)^10^, wavelet packet transform(WPT) combined with fuzzy neural network(FNN)^11^, MFCC and wavelet transform(WT) combined with K-nearest neighbors (KNN) classifier^15^, MFCC combined with self-attention Convolution neural networks(SACNN) and Logistic Regression (LG) classification algorithm^18^.

The bimodal DNN target recognition model proposed in Ref.^10^ consisted of two deep belief network, which processed the HHT features of acceleration and sound pressure signals respectively. And the transfer learning was employed to solve the problem of requiring a large number of samples for deep networks. In Ref.^11^, the sampled vibration signals were firstly decomposed by four-layer wavelet packet and reconstructed to obtain the total energy of signals in each frequency band as the feature of coal-gangue. Then, the constructed fuzzy neural network was applied to achieve coal-gangue identification. In Ref.^15^, firstly, features were extracted from phonocardiogram using three methods: time domain, frequency domain and time–frequency domain. Then, the features were optimized using genetic algorithm, and MFCC and WT were selected as classification features. Finally, KNN classifier was employed to evaluate the severity of tricuspid regurgitation. In Ref.^18^, MFCC was relied upon to create gender audio data, and the gender with different ages and different emotional states of speakers was identified by convolutional self-attention models and logistic regression.

This section focuses on two kinds of experiments, one is target recognition on noiseless dataset, and the other is target recognition on simulated site dataset. Tables 3 and 4 respectively list the F1-score comparison between other sound recognition methods and our method in these two kinds of experiments, and the comparison results adopt the fivefold cross validation method.

**Table 3:** F1-score (%) comparison with different methods on noiseless dataset.

State label	Methods				
	HHT + DNN^10^	WPT + FNN^11^	(MFCC + WT) + KNN^15^	MFCC + SACNN + LG^18^	Proposed MFCC + MBCNN
0	96.41 ± 3.29	82.07 ± 2.37	95.72 ± 2.94	93.62 ± 3.14	94.48 ± 2.48
1	95.05 ± 2.13	81.94 ± 3.07	94.73 ± 2.86	93.9 ± 2.93	94.61 ± 2.37
2	93.88 ± 1.23	80.92 ± 2.73	94.07 ± 1.56	97.92 ± 1.62	100 ± 0.00
3	93.27 ± 1.41	79.63 ± 2.38	95.84 ± 2.04	98.17 ± 1.27	100 ± 0.00
4	94.03 ± 2.62	81.53 ± 3.57	96.17 ± 2.99	100.0 ± 0.00	100 ± 0.00
5	93.47 ± 2.08	80.26 ± 1.86	95.94 ± 2.13	98.11 ± 1.07	100 ± 0.00
6	94.83 ± 1.95	81.25 ± 2.14	94.19 ± 1.82	100 ± 0.00	100 ± 0.00
Average	94.42 ± 2.10	81.09 ± 2.59	95.24 ± 2.33	97.39 ± 1.43	98.44 ± 0.69

**Table 4:** F1-score (%) comparison with different methods on simulated site dataset.

States	Methods				
	HHT + DNN^10^	WPT + FNN^11^	(MFCC + WT) + KNN^15^	MFCC + SACNN + LG^18^	Proposed MFCC + MBCNN
Coal	82.69 ± 3.71	75.19 ± 4.38	81.26 ± 2.84	89.05 ± 2.47	91.37 ± 1.04
Gangue	80.83 ± 4.25	77.24 ± 4.71	81.53 ± 3.17	88.11 ± 2.73	91.49 ± 1.26
Average	81.76 ± 3.98	76.22 ± 4.55	81.40 ± 3.01	88.58 ± 2.60	91.43 ± 1.15

According to Tables 3 and 4, it is not difficult to find that the traditional target recognition methods^10,11,15^ has poor F1-score performance on both noiseless dataset and simulated field dataset. The main reason is that the traditional methods can only extract or select a small number of features at different time scales or frequency bands. Especially for a single feature extraction method^11^, different classes may be similar, which reduces the classification accuracy. Ref.^10^ transferred knowledge from relevant data by transfer learning, which improves the weakness of limited labeled samples of deep neural networks. Ref.^15^ established an approximately optimal feature subset through feature selection to represent the original feature space, which improved the classification performance. Therefore, the recognition accuracy of HHT + DNN^10^ and (MFCC + WT) + KNN^15^ is higher than that of WPT + FNN^11^.

Refernce^18^ utilized the Self-Attention mechanism to search for regions of interest, optimized the ability of traditional CNN to process features, and achieved relatively good recognition accuracy. The proposed method in this paper can obtain 98.44% average F1-score on the noiseless dataset, especially in the case of labels 2–6, which can reach 100%. It is significantly superior to other methods on simulated field dataset and has more stable performance. This is mainly because MBCNN combined with MFCC smoothing can learn useful state classification features from different frequency scales, which not only retains the information from the original features, but also smooths the noise. It can be seen that the MBCNN based on MFCC smoothing proposed in this paper provides a method for target recognition in noisy environment.

### Applicability evaluation under diverse coal mines

**Figure 12:**
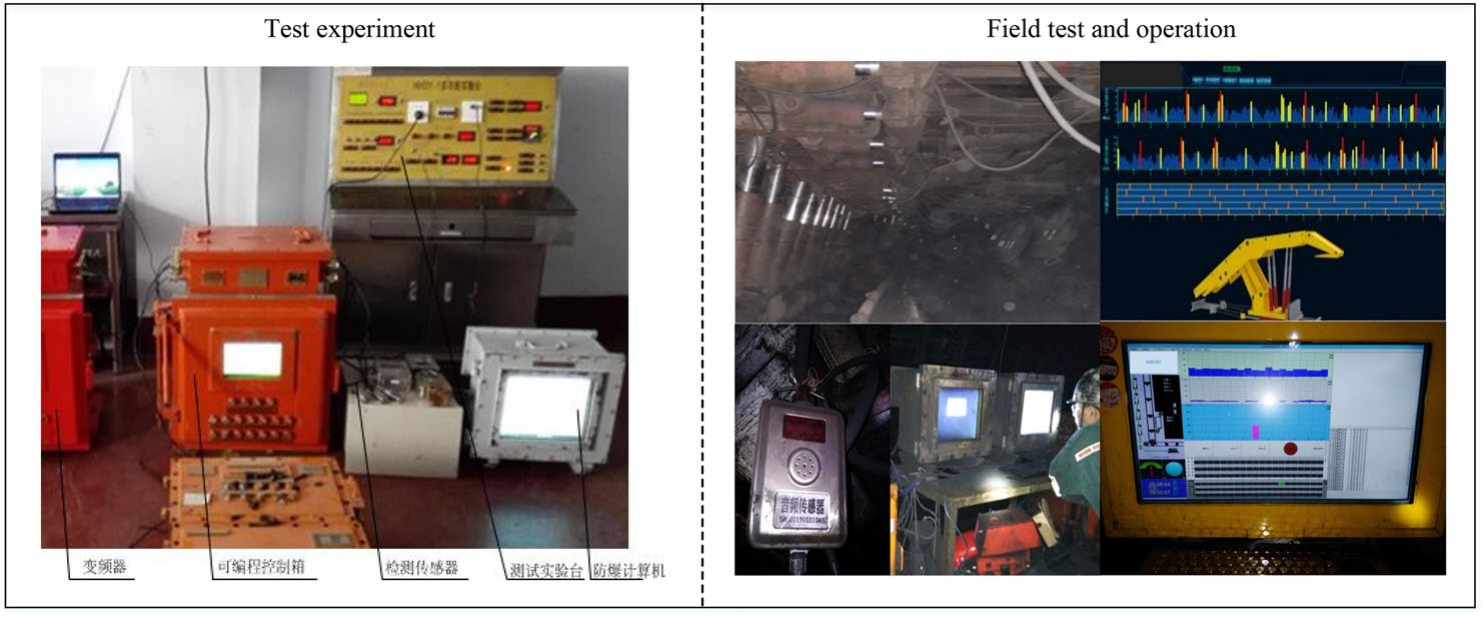
Data collection and operation in top coal caving production site.

The above experiments are based on the coal-gangue dataset in Shuiyu coal mine area. To further verify the transferability and applicability of the proposed method among different coal mines, a large number of on-site production datasets have been constructed by running self-developed coal caving automation systems (Fig. 12) in three coal mines with different geological conditions, namely Zhaojiazhai, Longgong and Shuiyu.

Based on three datasets of coal-gangue from Zhaojiazhai, Longgong and Shuiyu, F1-score over five folds was performed under single-branch (Model1), double-branch (Model2) and three-branch (Model3) CNN models to verify the applicability of the proposed method under different geological conditions. The experimental results are shown in Table 5.

**Table 5:** Performance (average value ± standard deviation) comparison between three coal mines.

Coal mine	Zhaojiazhai		Longgong		Shuiyu	
	Coal	Gangue	Coal	Gangue	Coal	Gangue
Model1	84.29 ± 3.05	83.61 ± 3.13	80.27 ± 2.87	81.06 ± 2.76	85.92 ± 3.58	86.74 ± 3.05
Model2	86.33 ± 2.07	85.59 ± 2.14	86.51 ± 2.31	87.47 ± 2.62	89.77 ± 2.54	88.92 ± 2.78
Model3	89.39 ± 2.23	90.29 ± 2.19	90.57 ± 2.04	90.84 ± 1.93	90.08 ± 1.85	91.12 ± 1.92

It is obvious from Table 5 that Model 3 (MFCC + MBCNN) approach gets the best overall performance of 91.08% *F*1-score. For each coal mine, Model 3 achieves the over 87% *F*1-score with a smaller standard deviation, so the classification results are more accurate. The average values of relative changes in *F*1-score for Model 1, Model 2 and Model 3 are 7.02%, 4.39% and 1.24%, respectively. The key difference in *F*1-score performance among the three models lies in the smoothing processing of MFCC features at different scales and the different feature mapping characteristics of multi branch convolutional neural networks. It can be seen that the method proposed in this paper can fully utilize the complementary information of the original features and denoised features, effectively suppress noise components, and improve the accuracy and applicability of the coal-gangue recognition system.

## Conclusions

In view of the noise induced by the operation of shearer, conveyor and other device mixed in the sound signal of coal or gangue in the process of top coal caving, a new multi-branch CNN structure based on MFCC feature processing was proposed to improve the recognition ability of coal-gangue in the noise environment. The following are the main conclusions of this study:

1. On the noiseless coal-gangue recognition dataset, the testing accuracy of the three-branch CNN model proposed in this paper is 0.79% higher than that of the traditional single-branch CNN. Although the accuracy is not much improved, the loss function and F1-score are significantly improved.
2. On the simulation site dataset with various noises, the testing accuracy of MBCNN is 6.0% higher than that of traditional CNN, the recognition accuracy rate of falling gangue state is 5.1% higher than that of double-branch CNN, and the stable convergence time is 35 epochs less than that of traditional CNN and 47 epochs less than that of double-branch CNN.
3. In addition, compared with other target recognition methods, the proposed method based on MBCNN and MFCC smoothing has better performance on both noiseless dataset and simulated site dataset in terms of F1-score.
4. Compared with the other two methods, Model 3 performs well in different geological experiments and has better adaptability.

In summary, the model constructed in this paper can provide effective feature information and good recognition accuracy in most cases. Therefore, as an extension of this work, we will try to use feature fusion methods to reduce the impact of noise on the correct recognition ratio of falling coal and gangue.

## Data Availability

The datasets used and analysed during the current study available from the corresponding author on reasonable request.
